# Anisotropic Silver Nanoparticles Gel Exhibits Antibacterial Action and Reduced Scar Formation on Wounds Contaminated with Methicillin-Resistant *Staphylococcus pseudintermedius* (MRSP) in a Mice Model

**DOI:** 10.3390/ani11123412

**Published:** 2021-11-30

**Authors:** Saengrawee Thammawithan, Oranee Srichaiyapol, Pawinee Siritongsuk, Sakda Daduang, Sompong Klaynongsruang, Nuvee Prapasarakul, Rina Patramanon

**Affiliations:** 1 Department of Biochemistry, Faculty of Science, Khon Kaen University, Khon Kaen 40002, Thailand; th_saengrawee@kkumail.com (S.T.); oranee_sr@kkumail.com (O.S.); pawinee.siri@kkumail.com (P.S.); somkly@kku.ac.th (S.K.); 2 Protein and Proteomics Research Center for Commercial and Industrial Purposes, Khon Kaen University, Khon Kaen 40002, Thailand; sakdad@kku.ac.th; 3 Division of Pharmacognosy and Toxicology, Faculty of Pharmaceutical Sciences, Khon Kaen University, Khon Kaen 40002, Thailand; 4 Department of Veterinary Microbiology, Faculty of Veterinary Science, Chulalongkorn University, Bangkok 10330, Thailand; nuvee.p@chula.ac.th

**Keywords:** *Staphylococcus pseudintermedius*, wound infection, antimicrobial resistance, alternative antimicrobial agent, nanotechnology, silver nanoparticles, scar reduction

## Abstract

**Simple Summary:**

Wound infection in animals with antimicrobial resistant bacteria, especially *Staphylococcus pseudintermedius*, plays an important role in the delay of wound healing. In this work, the antimicrobial and wound healing activities of gels containing anisotropic AgNPs were evaluated on wounds contaminated with Methicillin-resistant *Staphylococcus pseudintermedius* in a mice model. The results show that anisotropic AgNPs gel is effective in eliminating bacteria and preventing pus formation. Furthermore, anisotropic AgNPs gel exhibits improved collagen alignment that supports scar disappearance.

**Abstract:**

*Staphylococcus pseudintermedius* (*S. pseudintermedius*) infected wounds can cause seriously delayed wound healing processes in animals. Antimicrobial agents that have antimicrobial and wound healing efficacy have become an essential tool for overcoming this problem. In our previous study, anisotropic AgNPs have been reported to have antimicrobial efficiency against animal and human pathogens, and could be suitable as antimicrobial agents for infected wounds. Here, antimicrobial and wound healing activities of anisotropic AgNPs gels were assessed *in vivo*. BALB/cAJcl mice wounds were infected by Methicillin-resistant *Staphylococcus pseudintermedius* (MRSP). Then, antibacterial and wound healing activities were evaluated by bacterial cell count, wound contraction, digital capture, and histology. The results show that anisotropic AgNPs gels could eliminate all bacterial cell infected wounds within 7 days, the same as povidone iodine. Wound healing activity was evaluated by wound contraction (%). The results showed 100% wound contraction in groups treated with anisotropic AgNPs gels within 14 days that was not significantly different from povidone iodine and control gel without AgNPs. However, the digital capture of wounds on day 4 showed that anisotropic AgNPs gel prevented pus formation and reduced scar appearance within 21 days. The histology results exhibit improved collagen fiber alignment that supports scar disappearance. In conclusion, these results indicate that anisotropic AgNPs gels are suitable for treating infected wounds. The gel is effective in eliminating bacteria that supports the natural process of wound repair and also causes reduced scar formation.

## 1. Introduction

Bacterial infections can easily occur when animals have a wound that breaks the skin. Contamination at the wound site leads to a delay of wound healing and permits bacteria to spread to other organs. In animals in particular, wounds caused by bites are a common way that animals can get a bacterial infection, because their teeth are covered in bacteria [1]. Therefore, if a wound is caused by bites, they are usually more serious than they look and can easily become infected. Wound infections in animals are usually caused by opportunistic pathogens that are the normal microflora of the skin [2]. Similarly to *Staphylococcus aureus*, which is among normal flora on human skin [3], *S. pseudintermedius* is harmless in healthy individuals, but it is an opportunistic pathogen if an animal gets injured or sick [4]. These bacteria are one of the most common pathogens causing wound infection in animals [5]. 

*S. pseudintermedius* is a gram-positive cocci present in the skin and mucosa of animals [6]. Since it is an opportunistic pathogen in most animal species, it is an important pathogen in veterinary medicine. *S. pseudintermedius* has been isolated as a normal inhabitant of the skin, mucosa, nares, mouth, pharynx, forehead, groin and anus of healthy dogs, cats, horses, etc. [7,8]. In fact, 84.7% of all *S. pseudintermedius* isolates originate from canines [5]. *S. pseudintermedius* is a major cause of skin and ear infections, infections of other body tissues, cavities and wound infections in dogs and cats [6,9,10], and has become a pathogenic bacterium of veterinary concern, since a noted property of *staphylococci* is their ability to become resistant to antimicrobials [11,12]. It has been reported that up to 97.8% of Methicillin-resistant *S. pseudintermedius* (MRSP) isolates exhibit multidrug resistance (MDR) to three or more antibiotics routinely used in veterinary medicine [13,14]. Moreover, the bacteria is a zoonotic pathogen that can transmit to and infect humans [15,16]. Therefore, alternative antimicrobial agents to treat MRSP infected wounds are urgently needed in cases of wound infection in animals. 

Silver nanoparticles have been used as a topical agent in various formulations for several hundred years; they have been used in applications as diverse as photographics, conductive/antistatic composites, catalysts, biocides and as a wound treatment. The physical, chemical and biological properties of AgNPs depend on their size and shape [17], and in addition, stabilizers are also important to these properties [18,19]. Due to their nanoscale size and the various shapes of AgNPs, they exhibit good antimicrobial action against bacteria, fungi and viruses with low cytotoxicity to mammalian cells [20]. Furthermore, the wound healing activity of AgNPs has been reported in many research studies. AgNPs promote wound healing through anti-inflammatory properties, as indicated by reduced cytokine release, decreased lymphocyte and mast cell infiltration and inhibition of matrix metalloproteinase 2 and 9 [21,22]. In addition to good antimicrobial activity and wound healing properties, AgNPs are also suitable for application as antimicrobial and wound healing agents owing to: (1) Bacterial resistance to AgNPs is extremely rare, due to the presence of multiple bactericidal mechanisms [23]; (2) The fact that the surface of AgNPs can be easily modified [24]; (3) It is easy to synthesize using large scale, inexpensive, safe and reliable methods, including chemical, physical and biological methods [25]; (4) It is easy to synthesize in different shapes (spheres, rods, hexagons, triangles, tubes, plates, cubes) [26]. 

Owing to AgNPs having various sizes and shapes, they can be divided into two types following their shape; silver nanospheres are spherical in shape, whereas anisotropic AgNPs are non-spherical shapes of AgNPs, such as truncated triangle, hexagon, and rod. Anisotropic AgNPs show various physical, chemical, and biological properties, such as color of colloid solution, conductivity, antimicrobial action and cytotoxicity [27]. Due to their outstanding optical properties and electrical conductivity, anisotropic AgNPs are usually used for catalysts, sensors and imaging [28,29]. However, previous reports show anisotropic AgNPs also have a good antimicrobial effect [30,31]. Moreover, many reports display that the antimicrobial activity of anisotropic AgNPs as being higher than silver nanospheres [32]. Anisotropic AgNPs, such as truncated triangular, display the strongest antibacterial action, compared with spheres [33]. Another report has shown that silver nanoplatelets with triangular or circular shape are more toxic towards *S. aureus* than spherical and rod shapes [34]. In our previous study, anisotropic AgNPs, an appropriate mixture of nanospheres and nanoplates (i.e., circular disk, truncated triangle and hexagon), were synthesized via transformation of silver nanospheres with an optimum concentration of hydrogen peroxide. The anisotropic AgNPs have been presented with antimicrobial efficiency against animal and human pathogens with low cytoxicity to human cell lines [35]. Moreover, we have developed an antimicrobial gel containing AgNPs as an easy way to use an anisotropic AgNP-based antimicrobial formulation for topical use, such as for infected wounds. The gel can inhibit the growth of the bacterial pathogens and shows long-lasting protection against bacteria, compared with povidone iodine. Hence, in this study we evaluated antimicrobial and wound healing activities of gel containing anisotropic AgNPs toward wounds contaminated with MRSP in a mice model to assess the antimicrobial and wound healing efficacy of anisotropic AgNPs gel for alternative applications on infected wounds in animals as well as to expand their application to humans. 

## 2. Materials and Methods

### 2.1. Animals and Animal Care

The adult female BALB/cAJcl mice of 6 weeks old (17 to 25 g) that were used in this study were supplied by Nomura Siam International Co., Ltd., Bangkok Thailand. Animals were housed in specially designed cages with a size of 26.6 × 42.5 × 21 cm (6 mice per cage), in a room with a controlled temperature (23 ± 2 °C), 30–60% RH, and 12 h light/dark cycle, with specific pathogenic-free conditions, at the Northeast Laboratory Animal Center, Khon Kaen University. The experimental protocol was approved by the animal ethics committee of Khon Kaen University, Khon Kaen, Thailand (Animal Ethics KKU 3/61).

### 2.2. Bacterial Strain and Culture Media

MRSP MIC 411 (Exudate from dog wound) were kindly provided by the Department of Microbiology, Faculty of Veterinary Science, Chulalongkorn University. The bacteria were identified by the Vitek 2 Compact System (bioMérieux, Marcy l’Etoile, France). The oxacillin-resistant isolates (Minimum inhibitory concentration (MIC) ≥4 μg/mL) further underwent mecA gene existence test using the PCR method [36]. The bacteria was streaked on Mueller-Hinton agar (MHA) and incubated at 37 °C for 24 h. A colony was selected and inoculated with 5 mL of Mueller-Hinton broth (MHB, HiMedia Laboratories Pvt. Ltd., Bengaluru, India) at 37 °C overnight. Then, bacteria was sub-cultured in 5 mL of the same medium at 37 °C in a 180-rpm shaker-incubator for 3 h to yield a mid-logarithmic growth phase culture.

### 2.3. Anisotropic AgNPs Gel Preparation 

Tannic acid-stabilized anisotropic AgNPs were provided by our collaborator Prime Nanotechnology Co., Ltd. (Bangkok, Thailand) with a stock concentration of 100 mg/L. Anisotropic AgNPs were synthesized by transformation of silver nanospheres with an optimum concentration of hydrogen peroxide. For anisotropic AgNPs solution (a stock concentration of 100 mg/L) preparation, anisotropic AgNPs were prepared in sterile deionized water. In order to use anisotropic AgNPs in the form of a gel, poly(acrylic acid) (Sigma-Aldrich, St. Louis, MO, USA) was used to prepare the gel. Poly(acrylic acid) was chosen due its ease of preparation. Additionally, they have many advantages, such as good biocompatibility, keeping the wound moist and the ability to absorb many times their weight in water [37,38,39]. Briefly, 0.2 g poly(acrylic acid) was dissolved in 50 mL of sterile deionized water, followed by the addition 1 mL of glycerol (Sigma-Aldrich, St. Louis, MO, USA). After that, anisotropic AgNPs were added into the solution with final anisotropic AgNPs concentrations of 40 µg/mL (The minimum inhibitory concentration of anisotropic AgNPs against bacteria). Then, 0.2 g of triethanolamine (Sigma-Aldrich, St. Louis, MO, USA) was added to adjust pH and allow poly(acrylic acid) to form a gel. In the final step, sterile deionized water was added into the gel to make 100 g gel, and it was stirred well. The anisotropic AgNPs gel was stored at room temperature (27–30 °C) until use. The antimicrobial activity of anisotropic AgNPs gel was evaluated by diffusion test before application to mice. Briefly, the bacteria at an inoculum of 1 × 10^7^ CFU/mL was swabbed on an MHA plate. A hole with a diameter of 6 mm was punched aseptically with a sterile cork borer. Then, 0.04 mg/g of anisotropic AgNPs gel was filled into the wells at 0.2 g in triplicate and incubated at 37 °C for 24 h. Povidone iodine (Leopard medical brand Co., Ltd., Nakorn Pathom, Thailand) was used as the positive control, and the gel without AgNPs as the negative control. After 24 h of incubation, the inhibition zone was measured with the millimeter (mm) scale. 

### 2.4. Anesthesia and Wounding

All mice were anesthetized with intraperitoneal injection of a ketamine hydrochloride (PubChem, Bethesda, MD, USA) (90 mg/kg) and xylazine (Bimeda, Cambridge, ON, Canada) (10 mg/kg) saline cocktail [40]. The skin areas of the backs of the mice were cleaned with 70% ethanol (Siribuncha Co., Ltd., Nonthaburi, Thailand), povidone iodine (Leopard Medical Brand Co., Ltd., Nakhon Pathom, Thailand) and then 70% ethanol before wound creation. Wounds (8 mm in diameter) were made on the dorsal midline using sterile tissue scissors with curved blades. *S. pseudintermedius* MIC 411 were prepared to 10^7^–10^8^ CFU/mL in 5 mL of sterile Phosphate-Buffered Saline (PBS). Fifty microliters of the bacterial suspension were added to each wound bed immediately after wound surgery. After 2 days of wound creation, infected mice were divided into 3 groups (*n* = 6) including negative control (gel without AgNPs), positive control (povidone iodine; Leopard Medical Brand Co., Ltd.), and test group (anisotropic AgNPs gel). The mice had one agent applied once a day, every day for 14 days. 

### 2.5. Measurement of Wound Infection and Wound Size

Wound infection was performed using the serial dilution plate count method of bacterial load at the wound site on days 0, 4, 7, 10 and 14 before application of antimicrobial agents. Briefly, the wound sites were swabbed by cotton swab and kept in PBS buffer pH 7.4. Each sample solution was brought to 50 μL for a serial ten-fold dilution plate count (10^−1^–10^−8^ conc.) with sterile PBS buffer in triplicate. Then, 10 µL of each dilution was dropped on MHA and incubated at 37 °C overnight to count the bacterial colonies formed. In addition, all wounds were digitally photographed on days 1, 4, 7, 10, 14 and 21 to assess wound healing by the naked eye. Moreover, wound size was determined in diameter by using a vernier caliper on days 0, 4, 7, 10 and 14. Determinations were performed for wound area (mm^2^) using length and width measurements. Wound contraction was calculated using the following formula:

(1) $$Wound\;contraction\;\left(\%\right)=\frac{A_{0}-A_{t}}{A_{0}}\times 100$$

where *A*_0_ is the initial wound area and *A_t_* is the area of the wound at the time of image capture [41].

### 2.6. Histological Analysis

The animals were euthanized via CO_2_ inhalation on days 14 or 21, followed by immediately taking tissue from the wound area. The tissues of 0.5 × 0.5 mm^2^ were fixed in 10% neutral buffered formalin (NBF) (RCI Labscan Ltd., Bangkok, Thailand) for 24 h and then embedded in paraffin. Sections were cut at a thickness of 5 μm and stained with hematoxylin and eosin (H&E) (Sigma-Aldrich, St. Louis, MO, USA). Evaluations were made under a light microscope. 

### 2.7. Statistical Analysis

The bacteria load in the wound area and wound contraction data were expressed as the mean ± standard deviation. Analysis of variance with Tukey’s test was used for multiple comparisons. A *p*-value of less than 0.05 was considered significant.

## 3. Results

### 3.1. Antimicrobial Test of Anisotropic AgNPs Gel

**Figure 1:**
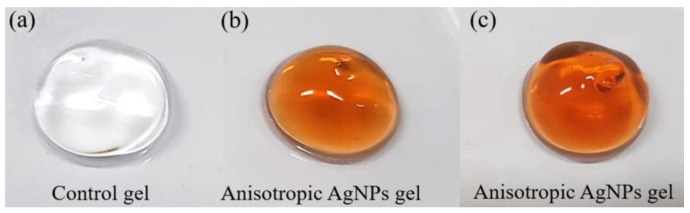
Anisotropic AgNPs containing formulation. Gel without AgNPs (**a**), Gel containing 0.04 mg/g of anisotropic AgNPs stored at room temperature (27–30 °C) for 24 h (**b**) and 1 year (**c**).

**Figure 2:**
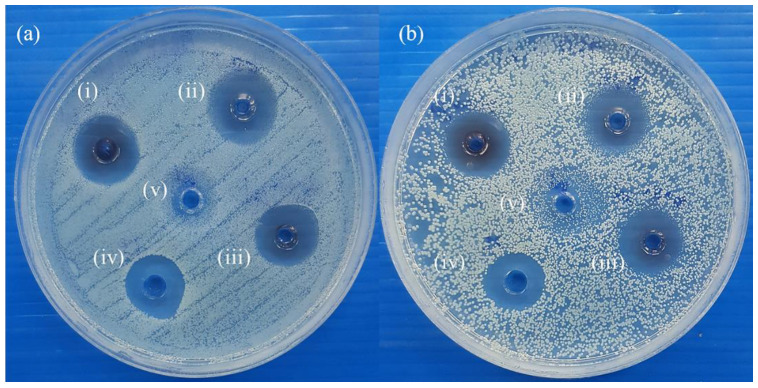
Antimicrobial evaluation of anisotropic AgNPs gel. Antimicrobial test of anisotropic AgNPs gel toward MRSP MIC 411 after preparation for 24 h (**a**), and after storage at room temperature for 1 year (**b**). Inset: anisotropic AgNPs gel (i–iii), povidone iodine (iv), control gel (v).

The characteristics of anisotropic AgNPs gel are shown in Figure 1. Control gel (without AgNPs) was colorless, while the gel contained anisotropic AgNPs was dark orange. Anisotropic AgNPs gel remained dark orange and was still stable after storage at room temperature after 1 year (Figure 1c). To evaluate antimicrobial activity of anisotropic AgNPs gel before in vivo testing, an antimicrobial screening test was performed by the diffusion method. As shown in Figure 2, anisotropic AgNPs gel presented an inhibition zone that indicated antimicrobial activity toward the bacteria. The antimicrobial activity of anisotropic AgNPs gel was tested after storage at room temperature for a year. The testing exhibited an inhibition zone at both 24 h and 1 year of storage. The average size of the inhibition zone had no significant differences at 16.37 ± 1.09 mm and 15.67 ± 0.98 mm when the gel was stored for 24 h and 1 year, respectively. This indicated that the anisotropic AgNPs gel still has antimicrobial activity even after storage for 1 year at room temperature.

### 3.2. Microbial Loads in Wounds

**Figure 3:**
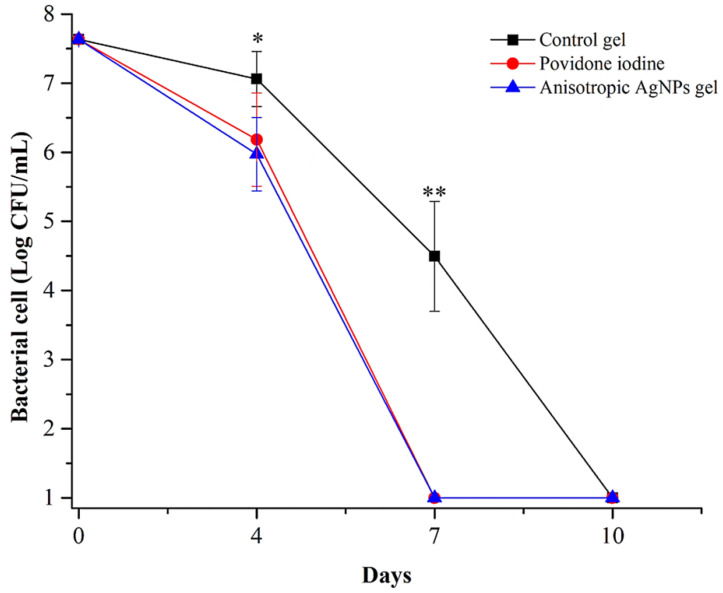
Bacteria load average in wound area. MRSP MIC 411 was loaded into the wound at 10^7^–10^8^ CFU/mL. The bacteria were evaluated on days 0, 4, 7 and 10. Data represent the mean value ± SD (error bar) of 6 mice (*n* = 6). * *p* < 0.05 and ** *p* < 0.001 are used as significant differences in comparison of the control gel with other groups.

To assess the antimicrobial activity of anisotropic AgNPs gel on wound infection, MRSP were infected with the initial inoculum of approximately 10^7^–10^8^ CFU/mL (Figure 3). The agents were applied after 48 h of infection. The results show that bacterial cells in the group treated with anisotropic AgNPs gel and povidone iodine were significantly less than the control group on day 4 and day 7 (* *p* < 0.05, ** *p* < 0.001). In the same way, these results show a continuous decrease of bacterial cells on day 0 to day 7 for groups that had anisotropic AgNPs gel applied and povidone iodine. On the other hand, the bacterial cells in the control group (Gel without AgNPs) continuously decreased from day 0 to day 10. These results exhibit that anisotropic AgNPs gel and povidone iodine can eradicate all the bacterial cells within 7 days, while the control group could eliminate all bacterial cells within 10 days. 

### 3.3. Wound Closure 

**Figure 4:**
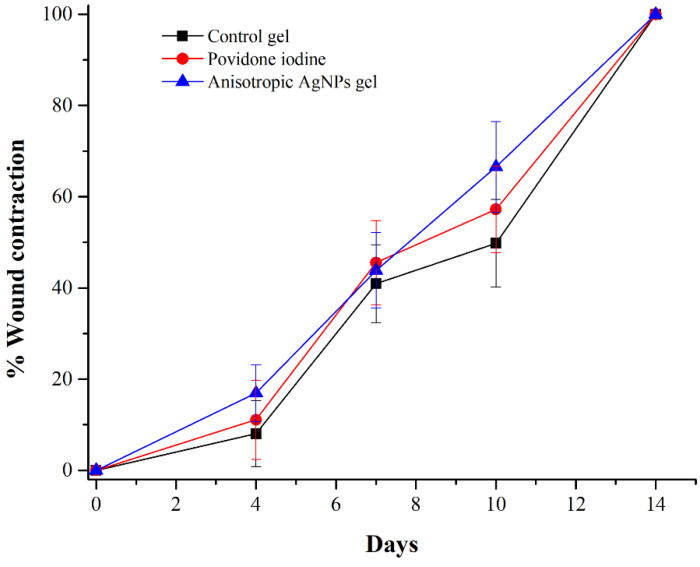
Wound contraction of experimental groups post treatment. Wound contraction (%) = ((wound area on day 0-wound area on day X)/(wound area on day 0)) × 100. Wounds were created 8 mm in diameter. After the wounds were created, they were left to infect with bacteria for 48 h. Control gel (without anisotropic AgNPs), povidone iodine, and anisotropic AgNPs gel were applied to the wounds on day 3 and continuously until the wounds had completely contracted. Wound size was measured on days 4, 7, 10, and 14 to calculate wound contraction. Data represent the mean value ± SD (error bar) for 6 mice (*n* = 6).

**Figure 5:**
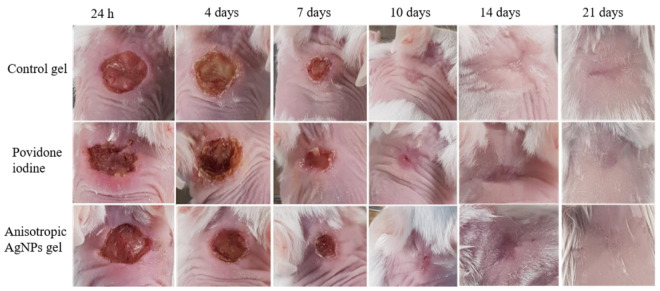
Photographs of wound repair at different times after excision of the wound model in mice. Effects of control gel (without anisotropic AgNPs), povidone iodine and anisotropic AgNPs gel on the healing of MRSP wound infection in mice. Representative images of mice from groups taken on days 1, 4, 7, 10, 14 and 21 after creation of the wound are shown. Mice were given a wound size 8 mm in diameter and bacterial cells added at 10^7^–10^8^ CFU/mL. After 48 h, the agents were applied to the wound.

We observed the wound healing activity of anisotropic AgNPs gel by quantitative measurement of the wound area on days 0, 4, 7, 10 and 14. The wound area on each day was compared with the initial wound area. Moreover, the wound healing was investigated by capturing digital photographs on days 1, 4, 7, 10, 14 and 21 of the experiment to evaluate wound features and scar formation. In Figure 4, wounds were made up to 8 mm in diameter, followed by adding bacterial cells at 10^7^–10^8^ CFU/mL. The results show that all groups had an efficiency of wound contraction, which appeared within 14 days. Within all groups, there was not a significant percentage of wound contraction on days 4, 7, 10 and 14. Similarly, digital photographs of wounds showed complete healing on day 14 for all groups. Interestingly, there was only one group that showed no visible scarring on day 21 (Figure 5) and that was the group treated with anisotropic AgNPs gel. From the foregoing, there was no difference in wound contraction observed among all groups within 14 days. However, scars were better in the group treated with anisotropic AgNPs gel. Moreover, it is noteworthy that on day 4 of wound creation, the control group revealed highly purulent wounds, while other groups did not show pus on wounds. 

### 3.4. Histology

**Figure 6:**
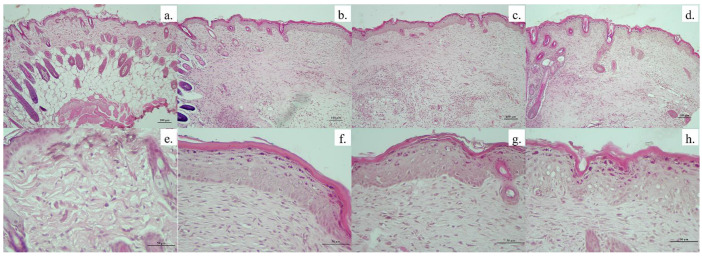
Photomicrographs of histological section of wound tissue of mice. Histology of wounds on day 14, stained with hematoxylin-eosin. Normal tissue (**a**,**e**), control gel (**b**,**f**), povidone iodine (**c**,**g**), anisotropic AgNPs gel (**d**,**h**) at scale bar of 100× (upper row) and 50× (lower row).

We evaluated histological sections of wound tissue on days 14 and 21 to support the healing properties of all groups. Hematoxylin and eosin were used to observe cells and tissue such as immune cells, fibroblast and collagen which implied wound status. Normal mouse skin was used as the control that indicated healthy skin. In Figure 6, normal skin showed an integrity of four layers, including epidermis (top layer skin), dermis (next to the top layer), sub-cutaneous fat (next to dermis) and muscle (deepest layer). In addition, there were some fibroblast cells inserted into the collagen fiber in the dermis layer. These are normal characteristics of mice skin. In other groups, including control gel (without AgNPs), povidone iodine and anisotropic AgNPs gel, mice tissue exhibited two layers of epidermis and dermis. They presented a lot of fibroblast cells on day 14. Moreover, the epidermis layer of all groups clearly appeared very swollen, when compared with normal skin. 

**Figure 7:**
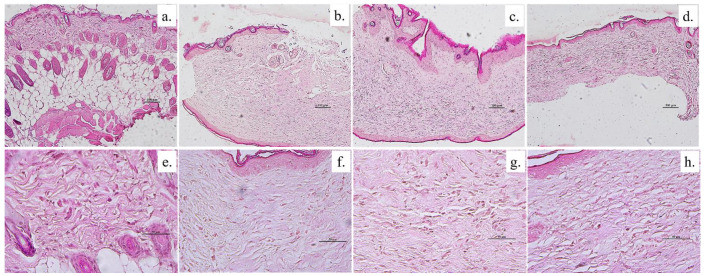
Photomicrographs of histological sections of mice wound tissue to evaluate collagen arrangement. Histology of wound tissue on day 21, stained with hematoxylin-eosin. Normal tissue (**a**,**e**), control gel (**b**,**f**), povidone iodine (**c**,**g**), anisotropic AgNPs gel (**d**,**h**) at scale bar of 100× (upper row) and 50× (lower row).

On day 21, as shown in Figure 7, the fibroblast cells in all groups exhibited a decrease when compared to day 14. Moreover, collagen fiber can be observed more clearly. Interestingly, the group treated with anisotropic AgNPs gel displayed collagen fiber that was the most obvious and the most similar to normal skin. In contrast, groups treated with control gel and povidone iodine exhibited too-tight collagen fibers when compared to normal skin. This collagen formation is also related to the amount of fibroblast cells that showed in each group (Figure 7e,f). Such results are consistent with photographs of mice skin without scars on day 21 in the group treated with anisotropic AgNPs gel (Figure 5). 

## 4. Discussion

*S. pseudintermedius* is an opportunistic pathogen in most animal species and is an important pathogen in veterinary medicine. Furthermore, the bacteria is a zoonotic pathogen that can transmit to and infect humans, and infection of the bacteria in wounds results in delayed wound healing. In this study, we made a gel that contained anisotropic AgNPs to overcome wound infection. In our previous study, we have shown that anisotropic AgNPs gels have antibacterial activities and prolonged antibacterial activity against animal and human pathogens in vitro [35]. Moreover, anisotropic AgNPs have antimicrobial activity as good as silver nanospheres, but with lower cytotoxicity [35]. Therefore, anisotropic AgNPs were used for this study. In this study, we reported in vivo capabilities of anisotropic AgNPs that appear to promote bacterial killing and improve wound healing, when infected with MRSP in a mice model of a skin wound.

The gel containing anisotropic AgNPs had antimicrobial efficiency and could be stored at room temperature for a year without reduction of antimicrobial efficacy. This result is the same as in a previous study, where AgNPs gel has a good antimicrobial activity and low cytotoxicity [22,42]. To assess the antimicrobial and wound healing efficacy of anisotropic AgNPs gels in vivo, BALB/cAJcl mice were used as a model of wound infection. Mice were given a wound that was infected by methicillin-resistant *S. pseudintermedius*. After 2 days of wound creation, antimicrobial agents were applied and antibacterial, as well as wound healing, activities were determined. The results of antibacterial tests show that the groups treated with anisotropic AgNPs gel can eliminate bacteria within 7 days, the same as povidone iodine, while the control group (gel without anisotropic AgNPs) needed 10 days to remove all bacteria from the wound. Owing to there being no previous reports on the use of AgNPs or AgNPs gel against *S. pseudintermedius* infected wounds in vivo, we can only compare the antibacterial efficacy with the reported results of pathogenic bacteria such as *S. aureus* that share several features with *S. pseudintermedius* [4]. We found that anisotropic AgNPs gel can eliminate *S. pseudintermedius* faster than those observed on *S. aureus* in the past. For example, AgNPs can eliminate all *S. aureus* cells infecting the wound on day 21 after wound induction [40]. In another study, *S. aureus* infected a burn wound, and it was found that AgNPs eliminated bacteria within 17 days after the induction of the burn wound [43]; indeed, the different efficiency at eliminating bacteria might be caused by the bacteria’s ability to cause disease. In this case, *S. pseudintermedius* MIC 411 mostly infects dog wounds and exhibits multidrug resistance [36]. These bacteria could contaminate a mice wound, but they might not cause infection similar to an infected dog wound. As shown in Figure 3, the mice’s immunity can eliminate bacteria within 10 days without applying antimicrobial agents. However, anisotropic AgNPs gel can support faster bacterial elimination. This point could be an advantage in the case of wound infection in which animals cannot eradicate bacteria by their immune system. 

Wound healing efficiency was assessed by the measurement of wound contraction. The results show that all groups were not different on wound healing efficiency, with complete wound contraction within 14 days. In general, mice wounds of size 8 mm in diameter can heal themselves within 14 days [44]. As observed in this study, infection of wound with *S. pseudintermedius* might not delay the wound healing process as shown by the antibacterial test (Figure 3), that mice can remove all bacterial cells within 10 days. The reason is that *S. pseudintermedius* might contaminate the wound, but they cannot replicate and disrupt the wound healing process. Therefore, mice can remove bacteria by their immune system and the results show that wound contraction was not different among all groups; however, even though the wound healing efficiency of anisotropic AgNPs gel was not different from the control group, it was noteworthy that on day 4 of wound creation in the groups treated with anisotropic AgNPs gel, images showed reduction of purulent wounds, similar to the groups treated with povidone iodine, while the control group showed highly purulent wounds. This indicates that anisotropic AgNPs gel prevents pus on wounds, which might be caused by reduction of bacterial cells and inflammation similar to povidone iodine [45]. Our results were similar to a previous report by P. F. Myronov et al., (2019), that AgNPs play an important role in cleaning wounds from purulence by decreasing the microbial contamination and the number of neutrophils and white blood cells [46]. In addition, there are many previous research reports that confirm the wound healing activity of AgNPs by reduction of inflammation through cytokine modulation and inhibition of MMP-2 and MMP-9 [22,47,48]. In another study, AgNPs reduced the levels of the prototypic cytokines, tumor necrosis factor (TNF) and interleukin (IL-6) [49]. Moreover, AgNPs have shown decreased mitochondrial function and induction of apoptosis or apoptosis-like changes of cell morphology [50,51,52]. However, these points could be studied further in the future to confirm the wound healing action of anisotropic AgNPs. 

Besides prevention of purulent wounds, it was clearly noticed in groups treated with anisotropic AgNPs gel that there was negligible scarring on day 21. That anisotropic AgNPs promote scarless wound healing, as found in our study, is similar to the study of Jun Tian et al. (2007); they have demonstrated that AgNPs act directly on dampening the process of inflammation, thus promoting scarless wound healing [47]. In addition, Jaya Jain et al. (2009) reported that, apart from antimicrobial activity, AgNPs inhibited MMPs and promoted scarless wound healing [22]. Moreover, V. Dhapte et al. (2014) proved that AgNPs presented scarless wound recovery without inflammation, because of effective cytokine modulation [53]. The scarless wound healing result corresponds to the histology result; mice given anisotropic AgNPs gel showed collagen fiber similar to normal skin on day 21. Collagen fiber is also important owing to the orientation and arrangement of collagen playing a key role during the remodeling phase, and consequently on the final scar appearance after wound closure. In this case, we could explain our result by the previous study of Karen H.L. Kwan et al. (2011), who pointed to the improvement of collagen properties of AgNPs that led to better fibril alignments in repaired skin, with a close resemblance to normal skin. AgNPs were predominantly responsible for regulating deposition of collagen, and their use resulted in excellent alignment in the wound healing process [54]. The collagen fiber alignment in this study supports scarless wound healing in the group treated with anisotropic AgNPs gel, and could be explained by scientific evidence since it had been previously studied. Nevertheless, the mechanism of collagen fiber alignment could not be elucidated in this study. 

**Figure 8:**
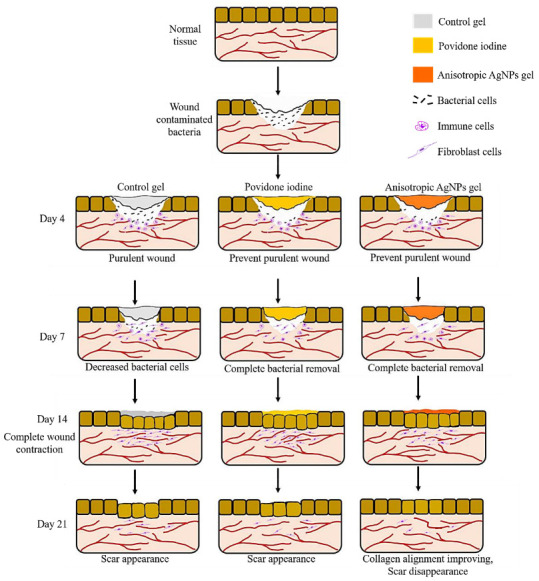
Wound healing process schematic of anisotropic ANPs gel. The anisotropic AgNPs gel reduced wound purulence after applying gel for 2 days, whereas the control gel exhibited high pus formation. After that, anisotropic AgNPs gel can remove all bacterial cells within 7 days and there is complete wound contraction within 14 days. Finally, improving collagen alignment by anisotropic AgNPs gel shows scar disappearance within 21 days.

Based on the present results, we summarize the wound healing process of anisotropic AgNPs gel against wounds contaminated with MRSP, as illustrated in Figure 8. For the wound after 2 days of applying agents, the number of bacteria in the groups treated with anisotropic AgNPs gel and povidone iodine are reduced, and the wound exhibits no purulence (Figure 3 and Figure 5), whereas the control group shows purulent wounds and exhibits more bacterial cells than other groups. The prevention of purulent wounds in the groups treated with anisotropic AgNPs gel and povidone iodine could be caused by a decrease in bacterial cells. On day 7, all the bacterial cells were eliminated in the groups treated with anisotropic AgNPs gel and povidone iodine, while the control group still had bacterial cell contamination (Figure 3). Next, the wound shows complete contraction within 14 days for all groups (Figure 4). This indicates that anisotropic AgNPs gel might not promote the wound contraction and quicken the healing rate. Interestingly, in the groups treated with anisotropic AgNPs gel, the wound shows scar disappearance as a result of collagen alignment within 21 days (Figure 6 and Figure 7), while the control group and povidone iodine group show scar formation. These imply that anisotropic AgNPs gel could affect the orientation and arrangement of collagen fiber. 

## 5. Conclusions

Wound infection in animals such as dogs and cats with antimicrobial resistant bacteria, especially *S. pseudintermedius*, plays an important role in the delay of wound healing. In a previous study, we showed that anisotropic AgNPs have antimicrobial efficacy against animal and human pathogens, and also anisotropic AgNPs have low toxicity to human cells *in vitro*. Therefore, antimicrobial activity and wound healing activity of anisotropic AgNPs *in vivo* were evaluated in this study. Based on the results, even though anisotropic AgNPs gel might not promote the wound contraction and quicken the healing rate, we have proved that anisotropic AgNPs gel can eliminate bacterial cells on infected wounds and reduce pus, as well as showing collagen alignment in repaired skin with a close resemblance to normal skin. This is important evidence to support scarless wound healing in mice, and this research indicates that anisotropic AgNPs gel could be suitable for treating infected wounds. Our findings support the potent antibacterial activities and scar reduction of anisotropic AgNPs gel as antibacterial agents in infected wounds. These gels can prevent the proliferation and colonization of opportunistic bacteria, and finally promote natural wound healing along with reduction of scar occurrence. 

## Data Availability

The data presented in this study are available upon request from the corresponding author.
